# Clinical relevance of circulating autoantibodies in idiopathic pulmonary fibrosis; A NAt hard to break

**DOI:** 10.3389/fmed.2022.964722

**Published:** 2022-08-08

**Authors:** Paraskevi Kirgou, Sotirios I. Sinis, Ilias E. Dimeas, Ilias C. Papanikolaou, Konstantinos Tatsis, Athena Gogali, Konstantinos I. Gourgoulianis, Dimitrios P. Bogdanos, Zoe Daniil

**Affiliations:** ^1^Department of Respiratory Medicine, Faculty of Medicine, School of Health Sciences, University of Thessaly, Biopolis, Larissa, Greece; ^2^Respiratory Medicine Department, Corfu General Hospital, Corfu, Greece; ^3^Respiratory Medicine Department, Faculty of Medicine, University of Ioannina, Ioannina, Greece; ^4^Department of Rheumatology and Clinical Immunology, Faculty of Medicine, School of Health Sciences, University of Thessaly, Biopolis, Larissa, Greece

**Keywords:** idiopathic pulmonary fibrosis, autoimmunity, antinuclear antibody, autoantibodies, interstitial lung disease, pulmonary function test (PFTs)

## Abstract

Patients with idiopathic pulmonary fibrosis are screened for circulating autoantibodies as part of the initial interstitial lung disease workup. Management of seropositive idiopathic pulmonary fibrosis is currently considered no different than that of lone idiopathic pulmonary fibrosis. Emerging data however suggest that the former may possess distinct characteristics in terms of pathophysiology, histopathology, prognosis and amenability to immunomodulation. In that context, the aim of our study was to evaluate the influence of autoantibody status on: (i) the decline of forced vital capacity; (ii) the decline of diffusing capacity of lung for carbon monoxide; and (iii) 3-year survival; in a cohort of 102 idiopathic pulmonary fibrosis patients. In a pilot sub-study, we also sought to evaluate whether changes in antibody status during disease course affect the aforementioned parameters by potentially reflecting activity of the autoimmunity component of the pro-fibrotic mechanism.

## Introduction

Idiopathic pulmonary fibrosis (IPF) is the most common form of progressive idiopathic interstitial pneumonia characterized by a specific type of radiological/histopathological pattern termed Usual Interstitial Pneumonia (UIP) (1). Paramount to the diagnosis of IPF is exclusion of interstitial lung disease in the setting of connective tissue disease (CTD-ILD) as UIP can be found in patients with systemic sclerosis, rheumatoid arthritis and polymyositis/dermatomyositis (1). The 2018 ATS/ERS/JRS/ALAT guidelines recommend routine screening for circulating anti-nuclear antibody (ANA), rheumatoid factor (RF), anti-cyclic citrullinated peptide antibodies (anti-CCP) and anti-myositis panel (1). Autoantibody positivity has been reported in 23–41% of IPF patients and ~8–10% of those with an initial diagnosis congruent with IPF will eventually develop overt CTD (2). Another portion of UIP cases may demonstrate clinical, histological and serological aspects of autoimmunity inconsistent with distinct CTD and are categorized as Interstitial Pneumonia with Autoimmune Features (IPAF) (3). In other words, a significant number of seropositive cases under the guise of IPF are likely to present with an autoimmunity phenotype/flare and be therefore rediagnosed as IPAF or CTD-ILD. On the other hand, the significance of circulating autoantibodies in patients with IPF that never fulfill criteria for IPAF or CTD-ILD remains controversial.

In light of the ineffectiveness of current immunomodulatory/immunosuppressive agents in IPF, the pathophysiological implications of autoimmunity have been markedly understated. Recent studies however concerning aberrant function of CD4+ and regulatory T cells and humoral autoreactivity against nuclear and cytoplasmic antigens in IPF have spurred novel interest on the paradigm (4). As a result there have been several publications contemplating the link between circulating autoantibodies and clinically impactful IPF endpoints such as: (i) patient survival, (ii) pulmonary function test parameters at diagnosis, (iii) pulmonary function test (PFT) parameter progression, (iv) acute exacerbation frequency or severity, and (v) response to treatment with antifibrotics or immunosuppression; albeit with conflicting outcomes (5–9). While it cannot be excluded that circulating antibodies are randomly autoreactive, it is entirely possible that they reflect an ongoing autoimmune process that participates in the mechanism culminating in pulmonary fibrosis. Considering the above, it would be prudent to explore whether autoantibody profiling denotes clinical phenotypes that could be more amenable to immunomodulation given the dismal prognosis of IPF and the urgent need for effective pharmacological intervention (10).

In this study we retrospectively assessed the frequency of an extensive panel of circulating autoantibodies in IPF patients belonging to three reference centers in Greece. We sought to perform associations between serological status and demographics, baseline PFT parameters, PFT parameter decline and 3-year survival. In a pilot sub-study the influence of seroconversion and autoantibody clearance on disease trajectory were investigated.

## Materials and methods

### Study population

The records of 102 patients diagnosed with IPF by an interdisciplinary care team (pulmonologist, rheumatologist, and radiologist) at the Outpatient ILD Clinics of Respiratory Medicine Department of University Hospital of Larissa, Respiratory Medicine Department of University Hospital of Ioannina and Respiratory Medicine Department of Corfu General Hospital between 2017 and 2020 were reviewed retrospectively. The diagnosis was based on the results of high- resolution computed tomography according to the 2018 ATS/ERS/JRS/ALAT guidelines (UIP pattern) (1). Demographics, pulmonary function tests and serum antibody titers were recorded.

### Serologic auto-antibodies profile

All patients were tested for serum autoantibodies at diagnosis and at regular visits after 12, 24, and 36 months. The results reported tests for conventional IgG ANA (antinuclear antibody), anti-ENA (extractable nuclear antigen), anti- SSA (Ro), anti- SSB (La), anti- RNP (ribonucleoprotein), anti- SM (Smith), anti- Scl70 (topoisomerase 1), anti-Jo1, antineutrophil cytoplasmic antibodies cytoplasmic and perinuclear (cANCA and pANCA, respectively), anti-dsDNA (double stranded DNA), anti-CCP (cyclic citrullinated peptide) and RF (rheumatoid factor). The titer of all serologic screening antibodies was considered positive if the result was above cut-offs recommended by the manufacturer. ANA testing was performed by conventional indirect immunofluorescence using HEp-2 as antigenic source (Euroimmun, Lübeck, Germany) and a test was considered positive at >1:80. ANCA testing was performed by indirect IFL using human neutrophils (Euroimmun) (>1:20). Positive tests by IFL were cross-checked by an independent diagnostician. All other tests were performed by conventional ELISAs or blot assays, as thoroughly described previously (Euroimmun). All positive tests were re-checked in duplicate (11).

### Pulmonary function tests

All patients underwent pulmonary function testing at baseline and at regular follow up every 12 months. Spirometry, diffusing capacity of lung for carbon monoxide (DLCO) and body plethysmography were recorded according to published guidelines (12). All the parameters were expressed in absolute terms and as a percentage of the predicted value. Gender, age and height were the variables used for calculating the predicted values for each PFT parameter (13).

### Statistical analysis

Results are given as mean (±Standard Deviation, SD) in normally distributed parameters and as median (±Standard Error, SE) in non-normally distributed values. Kolmogorov–Smirnov test was used to identify whether a continuous variable was normally or non-normally distributed. Normally distributed continuous indices were compared with Student's *t*-test or one-way Anova; non-normally distributed indices were compared *via* the Mann-Whitney-U and Wilcoxon test or Kruskal-Wallis test. Survival was reviewed by using Kaplan Meier analysis and hazard by implementing Cox Regression. Finally, Chi-square was used when testing categorical data. Data were analyzed using SPSS (IBM SPSS statistics version 25).

## Results

The follow up time after diagnosis was 3 years. The study involved 102 participants with a mean age of 71.8 years and a male/female ratio of ~4:1. The patients were subject to pulmonary function tests and had their blood drawn at diagnosis and at 12, 24,and 36 months post-diagnosis. A positive smoking history was obtained from 76 participants divided in 13 current smokers and 63 ex-smokers. The study population had a mean Forced Vital Capacity (FVC) of 2.67 ± 0.84 L (77.48 ± 19.22%) and a mean DLCO of 3.96 ± 1.53 mmol/min/kPa (48.20 ± 16.4%) at diagnosis (Table 1).

**Table 1:** Cohort demographic data and evolution of pulmonary function test parameters for seronegative patients and seropositive patients.

	**Seronegative patients**	**Seropositive patients**	***p*-value**	**Cohort**
Patient number	55	47	-	102
Age (mean ± SD)	72.40 ± 9.60	71.10 ± 9.43	>0.05	71.80 ± 9.50
Male	83.60%	76.60%	>0.05	80.40%
**Smoking status**				
Non	11 (20%)	15 (31.9%)	>0.05	26 (25.5%)
Ex	38 (69.1%)	25 (53.2%)	>0.05	13 (61.8%)
Active	6 (10.9%)	7 (14.9%)	>0.05	63 (12.7%)
**Autoantibodies**				
ANA	-	35 (74.5%)	-	-
RF	-	14 (29.7%)	-	-
Antigen specific	-	5 (10.6%)	-	-
**Pulmonary function trajectory**				
Baseline absolute FVC (mean ± SD)	2.66 ± 0.9	2.67 ± 0.75	>0.05	2.67 ± 0.84
Baseline FVC % predicted (mean ± SD)	77.66 ± 21.88%	79.02 ± 17.42%	>0.05	77.48 ± 19.22%
Baseline absolute DLCO (mean ± SD)	3.85 ± 1.6	4.09 ± 1.45	>0.05	3.96 ± 1.53
Baseline DLCO % predicted (mean ± SD)	45.92 ± 15.92%	51.01 ± 16.89%	>0.05	48.2 ± 16.4%
Total ΔFVC (mean ± SD)	−2.85 ± 14.1%	−7.73 ± 12.7%	>0.05	−5.26 ± 13.63%
Total DLCO (mean ± SD)	−14.45 ± 14.26%	−7.86 ± 12.42%	0.041	−14.45 ± 2.05%
Mean annual ΔFVC (mean ± SE/SD)	−1.23 ± 2.46%	−1.78 ± 4.01%	>0.05	−1.45 ± 0.61%
Mean annual ΔDLCO (mean ± SD)	−6.92 ± 6.81%	−3.73 ± 7.53%	0.042	−4.82 ± 7.41%
Clinically significant FVC decline	40 (72.7%)	37 (78.7%)	>0.05	77 (75.5%)
Year 1	10 (18.2%)	9 (19.1%)	>0.05	19 (18.7%)
Year 2	9 (16.4%)	10 (21.2%)	>0.05	19 (18.7%)
Year 3	7 (12.7%)	6 (12.8%)	>0.05	13 (12.7%)
Year 1+2	4 (7.3%)	4 (8.5%)	>0.05	8 (7.8%)
Year 1+3	3 (5.5%)	2 (4.3%)	>0.05	5 (4.9%)
Year 2+3	5 (9.1%)	4 (8.5%)	>0.05	9 (8.8%)
Year 1+2+3	2 (3.5%)	2 (4.3%)	>0.05	4 (3.9%)
Clinically significant DLCO decline	34 (61.8%)	28 (59.6%)	>0.05	62 (60.8%)
Year 1	12 (21.7%)	9 (19.2%)	>0.05	21 (20.6%)
Year 2	9 (16.3%)	9 (19.2%)	>0.05	18 (17.7%)
Year 3	4 (7.3%)	7 (14.9%)	>0.05	11 (10.8%)
Year 1+2	3 (5.5%)	1 (2.1%)	>0.05	4 (3.9%)
Year 1+3	3 (5.5%)	1 (2.1%)	>0.05	4 (3.9%)
Year 2+3	3 (5.5%)	0 (0%)	>0.05	3 (2.9)
Year 1+2+3	0 (0%)	1 (2.1%)	>0.05	1 (1%)

*Total ΔFVC or ΔDLCO = percent difference between absolute value at diagnosis and at 3 years post-diagnosis, Mean annual ΔFVC or ΔDLCO = mean value of percent decline occurring during year 1, 2, and 3 post diagnosis, Clinically significant FVC decline = annual decline ≥5% at least once during follow-up (max. 3), Clinically significant DLCO decline = annual decline ≥15% at least once during follow-up (max. 3). Patients were further stratified based on which year(s) clinically significant FVC or DLCO decline was observed*.

*SD, standard deviation; SE, standard error*.

Gender, smoking, age, baseline FVC, FVC percent decline during the first year following diagnosis, average annual percent FVC decline or total percent FVC decline over 3 years, average annual percent DLCO decline or total percent DLCO decline over 3 years were not associated with all-cause mortality. On the other hand, baseline DLCO (HR = 1.66. 95% CI 1.09–2.54 *p* = 0.018) and DLCO percent decline during the first year following diagnosis were associated with all-cause mortality in IPF patients (HR = 0.94 95% CI 0.89–1, *p* = 0.049).

It was found that 47 out of 102 (48%) enrolled subjects had detectable circulating autoantibodies at some point during the disease course. ANA was found in 35 patients, RF was found in 14 patients and the antigen-specific antibodies of the study panel were detected in 5 patients (Table 1). No statistical difference was observed in terms of baseline demographics and pulmonary function test values between seropositive and seronegative patients.

**Figure 1:**
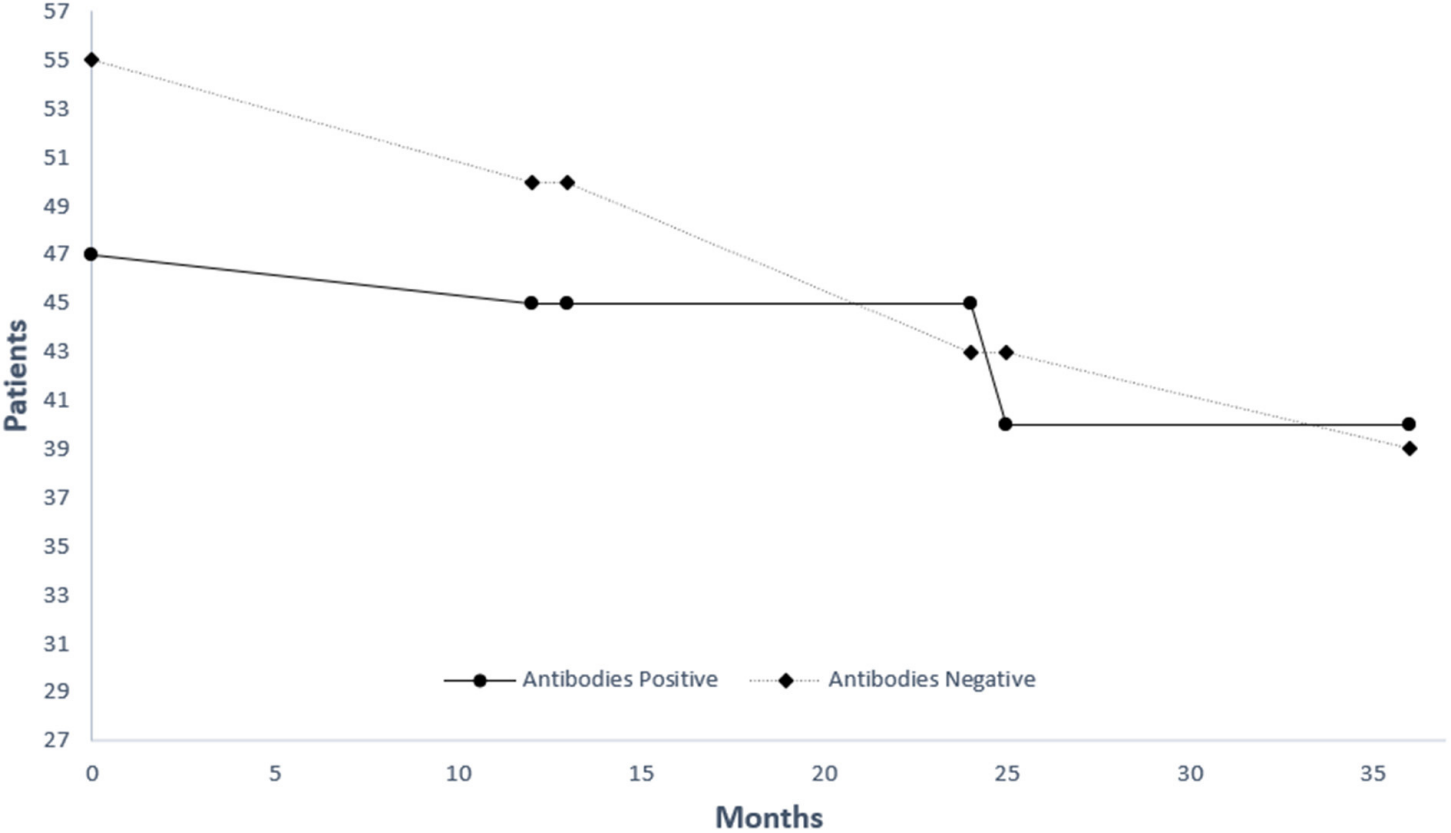
Kaplan-Meier estimate of survival for patients with (black line) and without (grey line) antibodies.

The rate of FVC decline was independent of autoreactivity status in this cohort (Table 1). On the other hand, all patients with circulating autoantibodies showed slower deterioration when assessed for changes in DLCO (Table 1). Autoantibody status was not associated with all-cause mortality in IPF patients although a tendency for benefit was observed for seropositive individuals (HR = 0.77. 95% CI 0.23–1.33) (Figure 1).

We speculated that circulating autoantibody temporal kinetics may define distinct disease phenotypes on the basis that innate immunity activity against self antigens may be an important avenue of fibrosis propagation in autoantibody positive IPF. Patients of: (i) Group A (*n* = 29) were persistently seropositive; (ii) Group B (*n* = 11) developed autoreactivity after the diagnosis; and (iii) Group C (*n* = 7) became seronegative as fibrosis progressed. The remaining patients were the 55 seronegative controls comprising Group D. However, no statistically significant differences were observed between groups A, B and C in terms of FVC and DLCO baseline values/trajectory or 3-year survival.

## Discussion

Our study contributes to the small but growing body of literature assessing the rate of decline in PFT parameters of IPF patients with and without circulating autoantibodies. This is the first report aiming to determine the implications of autoantibody kinetics in IPF. The results suggest that patients with circulating autoantibodies at some point during the disease course progressed slower in terms of DLCO compared to seronegative controls. There was a tendency for improved survival in seropositive participants but it was not statistically significant. Persistently seropositive patients were no different in terms of study endpoints compared to patients that developed seroconversion or those that became seronegative.

It remains unclear if circulating autoantibodies are more common in IPF patients compared to healthy individuals. In the publication of Lee et al., which is one of the few to address the issue, positive serology was found in 22% of IPF patients (*n* = 67) similar to that of internal controls (*n* = 52) (6). However, the lack of statistical significance becomes intriguing when taking into context that; (i) the IPF group had a higher proportion of males (50% males for the control group vs. 75% males for the IPF group); and (ii) healthy females are more likely to harbor circulating ANA.

Analysis and comparison of data regarding circulating autoantibodies in IPF patients is hindered by considerable heterogeneity between studies in critical parameters such as positivity cut-off values, population demographics, autoantibody selection and laboratory methodologies. At a low dilution of ≥1:40, Fischer et al. and Kang et al. reported ANA prevalence of 34 and 31.5% in their cohorts of 285 and 526 patients, respectively (8, 14, 15). We demonstrated that 48% of patients with IPF were seropositive for ANA, RF and/or antigen specific autoantibodies and that 34.4% had ANA ≥ 1:160, a level which is more clinically relevant. Two studies involving the same cut-off point showed a large discrepancy in terms of frequency with 41.4% out 58 and 16.8% out 386 individuals being positive, respectively (5, 16). Both research groups screened patients for antigen-specific autoantibodies such as ANCA, anti SSA/SSB, antiScl70 and antiJo-1, a strategy which moderates misclassification of patients as seronegative owing to untested markers of humoral autoimmunity. Nevertheless, no significant correlation between autoantibody presence and survival was found. It must be noted though that Moua et al. screened a portion of the population for antigen-specific autoantibodies which could result in false negative results (16). Even patients with ANA ≥ 1:320 and/or antigen specific autoantibodies showed no alterations in terms of life expectancy (17). The decline of PFT parameters during disease progression (annual absolute and percent predicted FVC or DLCO) remained unaffected in disagreement with our findings (17). However, after adjustment for previously established FVC and DLCO decline cut-offs (>5 and >15%, respectively) associated with survival, the influence of autoantibody positivity on DLCO becomes negligible in our study (Table 1) (18, 19). Taken together, the aforementioned body of evidence support the prevailing notion that CTD-related autoantibodies in IPF patients are probably inconsequential.

We did observe a statistically significant reduction in DLCO decline and Lee et al. reported longer transplant-free survival in seropositive individuals although both outcomes are of ambiguous clinical relevance (6). Extension of the follow-up period in our study could resolve the apparent contradiction, given the prognostic value of DLCO, and produce improvement of survival in line with the latter. It was recently shown by, Ghang et al. that autoantibodies as defined by the IPAF serologic domain and ANCA confer a favorable prognosis (7). Intriguingly, immunomodulation was beneficial to seropositive subjects which is consistent with the fact that circulating autoantibodies in IPF patients have been previously linked to CTD-like findings on lung histology (20). On the other hand, seronegative comparators showed greater 5-year mortality when treated (7). Parallels can be drawn with the study of Tzouvelekis et al. where combined emphysema and fibrosis patients with autoimmune markers showed better prognosis and abundant CD20+ B cell lymphoid follicles compared to those lacking autoreactivity indices (4). In agreement with the above, a recent pilot trial suggested that antibody reduction through plasma exchange, rituximab and intravenous immunoglobulin may quench acute exacerbations of IPF, a condition likely to be characterized by immunity perturbations found in classical autoantibody-mediated disorders (21). Within that context, we suggest that the clinical redundancy of circulating autoantibodies in IPF may need to be revisited.

Serial autoantibody measurements at follow-up visits for patients with an initial diagnosis most congruent with IPF has been proposed to monitor development of CTD given that disease presentation may be limited to pulmonary manifestations and the likelihood of misdiagnosis should be considered. For instance, in a Chinese study 25.1% of patients with ILD became seropositive after the initial diagnostic assessment (22). We sought to unravel additional value beyond CTD exclusion for monitoring levels of circulating autoantibodies in IPF. It is speculated that regular screening could identify patients developing activation of innate immunity against self antigens at some point during disease evolution (23). Given the different types of inflammation between rapid and slow progressors, changes in antibody status could represent a harbinger of the rate of functional decline and even response to immunosuppression (24). Although our exploratory trial did not show any difference in PFT trajectory and survival between groups A, B, and C, it is worthwhile to further delineate the implications of the dynamic inflammatory process during the course of IPF.

Our study has several limitations. Due to the size of the cohort, stratification based on antibody temporal kinetics produced sub-groups each with a small number of patients, thereby diminishing the strength of statistical analysis. Although we thoroughly screened every participant for an extensive panel of circulating autoantibodies, seropositive patients were not further classified based on autoantibody type considering that a very small minority had humoral autoreactivity markers besides ANA and RF. Therefore, the notion that certain CTD-related circulating autoantibodies may be of greater relevance in the setting of IPF management could not be explored in this study. Unfortunately, no data were recorded in terms of acute exacerbations precluding associations between deviant adaptive immunity responses against self-antigens and frequency or severity of episodes. We performed serial measurements of autoantibodies and regular rheumatologic consultations, however given the follow-up duration of 3 years; it cannot be excluded that some patients will eventually develop features consistent with CTD and were therefore misdiagnosed as IPF.

Large, prospective, multi-center studies involving meticulous serial measurements of standardized ILD-associated autoantibody arrays are required to unfold the dichotomy surrounding the role of circulating autoantibodies in IPF. To this end, the recent discovery of unidentified autoantibody bands by immunoprecipitation in IPF patients provides a new avenue to explore the immunological background of the disease and particularly that of acute exacerbation, when adaptive immunity perturbation may be more prominent (25). Even if detection of CTD-related circulating autoantibodies is unequivocally proven incidental in IPF patients, it is possible that novel autoantibodies with mechanistic, diagnostic, prognostic and therapeutic relevance await discovery.

## Data availability statement

The raw data supporting the conclusions of this article will be made available by the authors, without undue reservation.

## Ethics statement

Ethical review and approval was not required for the study on human participants in accordance with the local legislation and institutional requirements. The patients/participants provided their written informed consent to participate in this study.

## Author contributions

PK contributed to the conception, design of the study, and the acquisition of data for the work. SS contributed to drafting the work and revised it critically for important intellectual content. ID contributed to the conception, design of the study, and the acquisition and statistical analysis of data for the work. IP, KT, and AG contributed to the acquisition of data for the work and performed critical revision of the manuscript for important intellectual content. KG contributed to the conception, design of the study, and performed critical revision for important intellectual content. DB contributed to acquisition of data for the work and performed critical revision for important intellectual content. ZD contributed to the conception of the study, interpretation of data for the work, and performed critical revision of the manuscript for important intellectual content. All authors contributed to manuscript revision, read, and approved the submitted version.

## Funding

PK is a recipient of financial support in the context of a doctoral thesis (grant number MIS-5113934). The implementation of the doctoral thesis was co-financed by Greece and the European Union (European Social Fund-ESF) through the Operational Programme—Human Resources Development, Education and Lifelong Learning—in the context of the Act—Enhancing Human Resources Research Potential by undertaking a Doctoral Research—Sub-action 2: IKY Scholarship Programme for Ph.D. candidates in the Greek Universities.


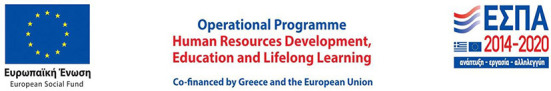


## Conflict of interest

The authors declare that the research was conducted in the absence of any commercial or financial relationships that could be construed as a potential conflict of interest.

## Publisher's note

All claims expressed in this article are solely those of the authors and do not necessarily represent those of their affiliated organizations, or those of the publisher, the editors and the reviewers. Any product that may be evaluated in this article, or claim that may be made by its manufacturer, is not guaranteed or endorsed by the publisher.

## Abbreviations

anti-CCP: anti-cyclic citrullinated peptide
anti-dsDNA: anti-double stranded DNA
cANCA, antineutrophil cytoplasmic antibodies: cytoplasmic
pANCA, antineutrophil cytoplasmic antibodies: perinuclear
ANA: antinuclear antibody
anti-RNP: anti-ribonucleoprotein
anti-SM: anti-Smith
anti-Scl70: anti-topoisomerase 1
CTD: connective tissue disease
ILD: interstitial lung disease
DLCO: diffusing capacity for carbon monoxide
anti-ENA: extractable nuclear antigen
FVC: functional vital capacity
IPF: idiopathic pulmonary fibrosis
IPAF: interstitial pneumonia with autoimmune features
RF: rheumatoid factor
PFT: pulmonary function test.
